# Effect of Two Meal Replacement strategies on Cardiovascular Risk Parameters in Advanced Age Patients with Obesity and Osteoarthritis

**DOI:** 10.3390/nu12040976

**Published:** 2020-04-01

**Authors:** Juan José López-Gómez, Olatz Izaola-Jauregui, David Primo-Martín, Beatriz Torres-Torres, Emilia Gómez-Hoyos, Ana Ortolá-Buigues, Miguel A. Martín-Ferrero, Daniel A. De Luis-Román

**Affiliations:** 1 Servicio de Endocrinología y Nutrición, Hospital Clínico Universitario de Valladolid, Av. Ramón y Cajal, 3, 47003 Valladolid, Spain; olatzizaola@yahoo.es (O.I.-J.); bsdavid_primo@hotmail.com (D.P.-M.); beatriztorrestorres@hotmail.com (B.T.-T.); emigomezhoyos@hotmail.com (E.G.-H.); anaortola@hotmail.com (A.O.-B.); dadluis@yahoo.es (D.A.D.L.-R.); 2 Instituto de Endocrinología y Nutrición (IENVA), Universidad de Valladolid, Av. Ramón y Cajal, 3, 47003 Valladolid, Spain; 3 Servicio de Traumatología, Hospital Clínico Universitario de Valladolid, Av. Ramón y Cajal, 3, 47003 Valladolid, Spain; miguelangel.martin.ferrero@uva.es

**Keywords:** obesity, osteoarthritis, meal replacement diet, cardiovascular risk

## Abstract

Background and aims: Meal replacement diets consist of replacing one or more meals with an artificial nutritional supplement. The objective of this study was to compare the effect of one against two meal replacement strategies on body composition and cardiovascular risk parameters in patients with obesity. Methods: A randomized clinical trial was designed with a modified hypocaloric diet with an artificial nutritional preparation replacing one or two meals for three months in patients with obesity and osteoarthritis pending orthopedic surgery. An anthropometric evaluation and a measurement of the body composition were done with bioelectrical impedance measurement at the beginning and at three months. Results: A total of 112 patients were recruited. Fifty-two patients (46.4%) were randomized to one replacement and 60 patients (53.6%) to two meal replacements. Eighty-one patients (72.3%) were women, and the average age was 61 (11.03) years. The percentage of weight loss at three months was 8.27 (4.79)% (one meal replacement: 7.98 (5.97)%; two meal replacements: 8.50 (3.48)%; *p* = 0.56). A decrease in fat mass measured by the fat mass index (FMI) was detected (one meal replacement: −2.15 (1.45) kg/m^2^ vs. two meal replacements: −2.78 (2.55) kg/m^2^; *p* > 0.05), and a relative increase in fat-free mass was observed (one meal replacement: +3.57 (4.61)% vs. two meal replacements: +2.14 (4.45)%; *p* > 0.05). A decrease in HOMA-IR, systolic blood pressure (SBP), and total cholesterol was observed in both groups without differences between them. Conclusions: The substitution strategies of one or two meal replacements were effective in weight loss and fat mass decrease without differences between the two groups. An improvement in lipid parameters, glycemic control, and systolic blood pressure was observed without differences between strategies.

## 1. Background 

Obesity is the most prevalent metabolic disease in the developed world and is one of the main causes of morbidity and mortality. Obesity has been defined as the greatest risk factor for the development of noncommunicable diseases (diabetes, cardiovascular disease, and cancer). In addition, obesity is considered along with high blood pressure, smoking, high blood sugar, and physical inactivity as the main risk factors for global mortality [1].

In the patient with advanced age, there is a redistribution of body composition with a progressive decrease in lean mass and an increase in fat mass [2]. This concept is called sarcopenic obesity. This pathology is characterized by a loss of muscle in the development of obesity, which is common in the elderly patient or in those patients with obesity associated with severe diseases. The genesis of this disease is related to the muscle damage caused by inflammatory mediators in the environment of a vicious circle of progressive physical inactivity that increases adipose tissue and obesity-related pathologies, which in turn increase this inactivity [3].

The pathological entity most strongly associated with obesity is metabolic syndrome, which is defined as an association of pathologies (central obesity, hypertension, dyslipidemia, and impaired carbohydrate metabolism) that increase the risk of the development and progression of cardiovascular disease and type 2 diabetes mellitus (DM2) [4]. The effect of weight loss on cardiovascular risk influences the main determinants of metabolic syndrome (hypertension, dyslipidemia, and DM). In this way, a weight loss of 5% improves these cardiovascular risk factors [5].

The optimal treatment of obesity includes three pillars: diet, exercise, and behavioral modification. Losing and maintaining weight in a healthy way are the goals of any obesity treatment. In the aged patient, treatment is especially complicated due to comorbidities, frailty, sarcopenia, functional limitations, and mobility, as well as the social environment. Therefore, it is necessary to calibrate the interventions performed on this patient properly to avoid the loss of muscle and bone mass [6].

Meal replacement diets consist of replacing one or several meals with an artificial supplement. They are based on the control of caloric and nutrient intake in an artificial manner. The increase in involuntary intake of natural foods in conventional hypocaloric diets is avoided, both due to the error of the measure itself and the patient’s error. The effect on weight loss is relative and is conditioned by the short duration of these studies. In a meta-analysis that compared the effect of substitution diets of one or two meals with conventional diet, it was observed that at three months, the weight loss was greater in the replacement diet group than in the usual diet group. The percentage of patients who achieved a decrease > 5% was 72% in patients with a replacement diet and 34% in conventional patients [7]. On the other hand, in a meta-analysis comparing different types of diets, a loss similar to that of other types of diets that included intensification of follow-up was observed [8]. There are other more recent randomized clinical trials that corroborate the data referred to in previous meta-analyzes, but there are also different studies that have not found a difference compared to conventional diets [9]; although, in one of them, there was a change in body composition with a greater loss of fat mass with respect to lean mass [10]. 

Improvement of pain and functional disability is the main objective of the patient when considering a surgical treatment of osteoarthritis. Weight loss through conventional diet therapy has a positive effect on knee osteoarthritis [11]. However, due to physical limitations, this weight loss is discrete and can impair muscle mass [12]. An alternative to conventional dietary treatment in the surgical patient is meal replacement diets. It has been shown that this type of diet in patients with osteoarthritis results weight loss and an improvement in quality of life [13].

Given the difficulty of carrying out this type of meal replacement diet and the contradictory data of the existing evidence, it is necessary to evaluate the effect of this type of diet in different populations. Most previous studies of meal replacement used the substitution of two meals (lunch and dinner) [7,8]. This strategy is more difficult to adhere to, and many patients only replace one of these two meals in a long follow-up [14]. It would be interesting to compare the effect on weight, body composition, and cardiovascular markers of a strategy with two meal replacements versus one meal replacement a day.

The purpose of this study was to compare the efficacy of one against two meal replacements on weight loss, body composition, and cardiovascular risk factors in aged patients with osteoarthritis.

## 2. Methods

A randomized clinical trial with two branches was designed with a hypocaloric diet modified with a nutritional supplement (Vegestart ®) with replacement of one or two meals for 3 months in patients with obesity and osteoarthritis pending orthopedic surgery.

### 2.1. Scope

The study was conducted in patients with obesity belonging to the health area of Valladolid East. These patients were referred from the Traumatology Service to the Endocrinology and Nutrition Service of the Clinic Hospital of Valladolid for weight loss prior to orthopedic knee, hip, and/or spine surgery between January 2017 and December 2019.

The sample size was calculated, taking into account that, in order to detect differences of 5 kg of weight loss, in a population with an average weight of 85–90 kg and a deviation of 5–6 kg, assuming a power of 90%, an alpha error of 5% and a 5% loss rate would require at least 50 patients per branch.

Inclusion criteria: Patients with Obesity Grade I or higher (BMI > 30 kg/m^2^). Age > 18 years. Degenerative osteoarthritis (knee, hip, and/or spine) pending orthopedic surgery. Exclusion criteria: Ingestion of more than two alcoholic beverages per day. Malignant tumor pathology. Severe psychiatric pathology. Severe or terminal renal impairment (Stage IV or higher (creatinine clearance < 30 mL/min)). Refusal to participate in the study and/or non-compliance with informed consent.

All participants provided informed consent to the protocol approved by the local ethical review board of the Valladolid University Clinical Hospital (HCUVA) Ethics Committee. This study was registered in the clinical trial registry of HCUVA and University of Valladolid with the code FUNGE 061/140242. All procedures performed in studies involving human participants were in accordance with the ethical standards of the institutional and/or national research committee and with the 1964 Helsinki declaration and its later amendments or comparable ethical standards.

### 2.2. Intervention

After signing the informed consent and the inclusion of the patient in the study:

- A fasting baseline analysis, a baseline anthropometric assessment, was performed, and a measurement of body composition was performed using a bioelectrical impedance measurement.
- Patients received nutritional education and were randomized into two groups: a hyperproteic low-fat hypocaloric diet with a meal replacement with a hyperproteic normocaloric artificial nutritional supplement (Vegestart ®); a hypocaloric hyperproteic low-fat diet with a replacement of two meals with a hyperproteic normocaloric artificial nutritional supplement (Vegestart ®). A different diet was assigned to men and women. The diet in both groups was structured into 5 meals (breakfast, mid-morning, lunch, snack, and dinner). The lunch and/or dinner were replaced by an artificial nutritional supplement called Vegestart ® (200 mL bottle: 200 kcal caloric content; protein: 15.4 g (31% total caloric value (VCT)); lipids: 5.2 g (23% VCT); carbohydrates: 21 g (42% VCT)). The characteristics of the macronutrient composition of both diets are shown in Table 1. The composition of all the oral nutritional supplements used in meal replacement diets are regulated by the European Commission Directive 98/6/CD, which is included in the Spanish legal system by the “Real Decreto 1430/1997”.
- The patients were reevaluated three months after the start of the intervention with anthropometry, a measurement of body composition, and a fasting baseline analysis.

### 2.3. Variables

The variables studied were measured in the baseline situation and three months after starting the intervention. 

#### 2.3.1. Demographic Variables

The age of entry into the study and the sex of each subject were recorded. On the other hand, if they exercised or not, the hours dedicated to this exercise per week, and the intensity of this exercise were determined. A structured questionnaire to measure exercise was not used; this was collected by a clinical interview. 

#### 2.3.2. Clinic Variables

An interview with the patient was carried out with an exhaustive history exploring the different clinical variables. Among these were those related to the patient’s pathological history: cardiovascular risk factors: diagnosis of diabetes mellitus and type, arterial hypertension, and dyslipidemia (hypercholesterolemia and hypertriglyceridemia). Joint pathology: diagnosis of osteoarthritis of the knee, hip, and/or lumbar spine. Systolic (SBP) and diastolic blood pressure (DBP) was determined by averaging two measurements after a 10 minute break with a mercury sphygmomanometer (Omron, LA, USA). The units in which it was measured and expressed were millimeters of mercury (mmHg). 

#### 2.3.3. Anthropometry

The anthropometric assessment of the subjects was carried out by determining the weight, height, and body mass index (BMI); as well as waist and hip perimeter. 

The weight was measured without clothes with an accuracy of ± 0.5 kg, using a manual scale up to the nearest 0.1 kg (Seca, Birmingham, United Kingdom). Height was measured with the patient in an upright position to the nearest centimeter, using a stadiometer (Seca, Birmingham, United Kingdom). The body mass index (BMI) was calculated using the formula: weight (kg)/height^2^ (m^2^). The weight loss percentage (%WL) was used to assess the relative weight difference. It related the difference between the weight before and after the intervention with the initial weight according to the following formula: (weight after intervention (kg)/initial weight (kg)) × 100).

#### 2.3.4. Body Composition

In all the subjects of the study, a bioelectrical impedance analysis (BIA) was performed to estimate resistance, reactance, phase angle, total body water (TBW), intracellular water (IW), extracellular water (EW), fat-free mass (FFM), fat mass (FM), calorimetry, and basal metabolic expenditure. These measurements were made before the start of the dietary intervention and three months after it. The BIA was performed on all subjects after a fast of at least five hours, according to the manufacturer’s instructions. Given that it may be influenced by the degree of hydration, subjects were warned that they could not exercise or drink alcohol within 48 hours prior to the test [15]. This was determined by a single frequency tetrapolar equipment in recumbency. An alternating current of 0.8mA at 50 kHz produced by a calibrated signal generator (Biodynamics Model 310e, Seattle, WA, USA) was used and applied to the skin using adhesive electrodes placed on the back of the right hand and foot. The electrical parameters of resistance (R) and reactance (Xo) were directly assessed, and the alpha phase angle and impedance (Z) were estimated. Body composition was estimated, and vector impedance was assessed with Biva ® and Bodygram ® software. The fat-free mass index (FFMI) (kg/m^2^) calculated using the formula FFM (kg)/(height × height) (m^2^) was also determined.

#### 2.3.5. Biochemistry

##### Lipid Profile

Blood samples (serum and whole blood) were taken from each of the subjects under fasting and baseline conditions, and the determinations were made in the Clinical Analysis laboratory of the University Clinical Hospital of Valladolid. All determinations were analyzed on the Cobas Hitachi platform (Roche Diagnostics GmbH, Mannheim, Germany), and the calculation of LDL-cholesterol using the Friedewald Formula LDL-cholesterol = total cholesterol-HDL-cholesterol-triglycerides/5 (in mg/L) or triglycerides/2.1 (in mmol/L) if triglycerides < 250 mg/dL. 

##### Glucose Metabolism

Plasma glucose levels were determined by an automated glucose oxidase method (glucose analyzer 2, Beckman Instruments, Fullerton, California). Insulin was measured by enzymatic calorimetry (insulin, WAKO Pure-Chemical Industries, Osaka, Japan). Insulin sensitivity was calculated using the “homeostasis model assessment” (HOMA-IR) that was calculated using the following values ((glucose (mg/dL) × 0.05) x insulin (mUI/L))/ 22.5. The interpretation of HOMA-IR was performed according to HOMA 1-IR by Matthews et al. [16]. A HOMA-IR value greater than 2.5 was considered as an indicator of insulin resistance.

### 2.4. Statistical Analysis 

The data were processed using the SPSS statistical package (SPSS for Windows Version 15.0, 2008 SPSS INC, Chicago, Ill, USA). Continuous variables were described as the mean (SD) in the case of a normal distribution or as the median and interquartile range (P25–P75) if the distribution was non-normal. Qualitative variables were described by absolute and relative frequencies (percentages). Data were collected in tables and represented in the most appropriate graphs for each type of variable (bar chart for qualitative variables, frequency histogram for quantitative variables). To study the association between qualitative variables, the Chi-squared test was used, with Yates correction and Fisher’s exact test when the conditions required it. In the case of quantitative variables, the Kolmogorov–Smirnov test (when the size was greater than 30) and the Shapiro–Wilk test (when the size was less than 30) were used to determine the normality of the distributions. To study the differences between independent means, the parametric or non-parametric statistical tests required by the application conditions (Student’s *t* or Mann–Whitney’s U in the case of two categories) were used. To study the differences between paired variables, statistical tests were used: Student’s *t* for paired variables (normal variables) and Wilcoxon signed ranges test (non-normal variables). Finally, the relationship between quantitative variables was analyzed by Pearson’s correlation tests (in parametric conditions) or Spearman (under non-parametric conditions). The level of significance was conventionally set at *p* ≤ 0.05.

## 3. Results

**Figure 1:**
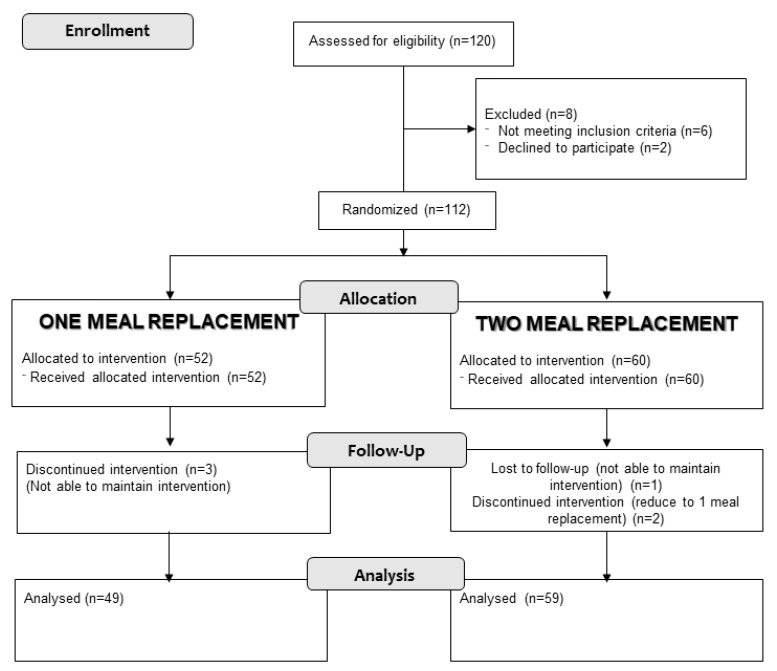
Flowchart.

A total of 112 patients were recruited. Fifty-two patients (46.4%) were randomized to one meal replacement group and 60 patients (53.6%) to the two meal replacement group. Three patients (2.7%) left the study due to inability to follow the dietary pattern, and three patients (2.7%) in the group of two meal replacements switched to the one meal replacement group by their own decision (Figure 1). Eighty-one (72.3%) patients were women, and the average age of the patients was 61 (11.03) years without a difference between both randomization groups in any of the variables (Table 2).

The main location of the osteoarthritis of the patients was the knee (79.5% of the patients). Regarding cardiovascular risk factors, 9 patients suffered from type 2 diabetes mellitus (8%), 10 patients had some type of dyslipidemia (8.9%), and 56 patients suffered from hypertension (50%). There were no differences in relation to these pathologies between the two groups (Table 2).

### 3.1. Anthropometry

Weight, waist, and hip data were analyzed. No differences were observed between both treatment groups at the beginning of the intervention. 

**Figure 2:**
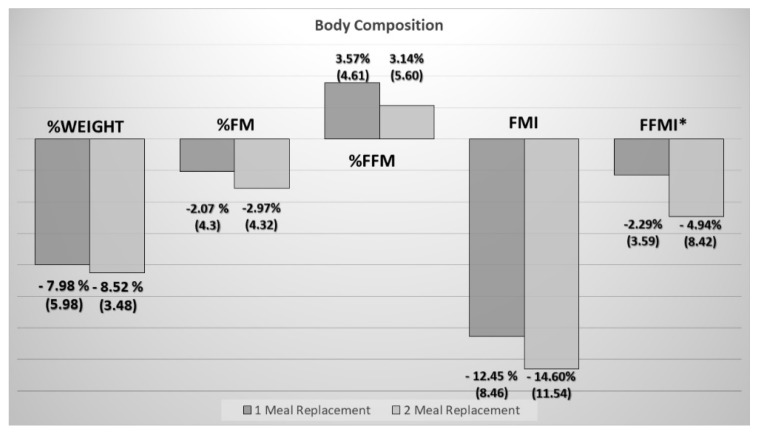
Comparison of changes over three months (percentage of change in the Y axis) in anthropometry and body composition between two groups. %FM: percentage fat mass from total weight; %FFM: Percentage Fat-Free Mass from total weight; FFMI: Fat-Free Mass Index; FMI: fat mass index (* *p*-value < 0.05).

Three months after the start of the intervention, a percentage of weight loss of 8.27 (4.79) % was observed without significant differences between the two treatment groups (Figure 2).

A significant decrease in waist diameters was observed in both groups, while only a significant decrease in hip diameter was observed in the substitution group of two meals (Table 2). There were no significant differences between the groups or waist (two meal replacements: 6.52 (6.29) cm vs. one meal replacement: 4.88 (16.29) cm; *p* = 0.48) or hip (two meal replacements: 6.79 (6.79) cm vs. 1 meal replacement: 2.2 (19.84) cm; *p* = 0.09) circumference.

### 3.2. Body Composition

No differences were observed in the relative percentage of the components of body composition (fat mass or fat-free mass) at the beginning of the intervention. There were no differences in the fat-free mass index (Table 2).

When analyzing body composition by impedance measurement, a decrease in fat mass measured by the fat mass index and fat-free mass measured by the fat-free mass index (FFMI) was detected in both groups. Although, a relative increase in the fat-free mass with respect to the fat-mass compartment was observed through the measurement of the percentage of fat-free mass and the fat-free mass/fat mass index (Table 3).

A greater deterioration in fat-free mass was observed in patients who consumed two supplements (Figure 2); although there was no significant difference in the relative change of the compartments in percentage (Figure 2). 

**Figure 3:**
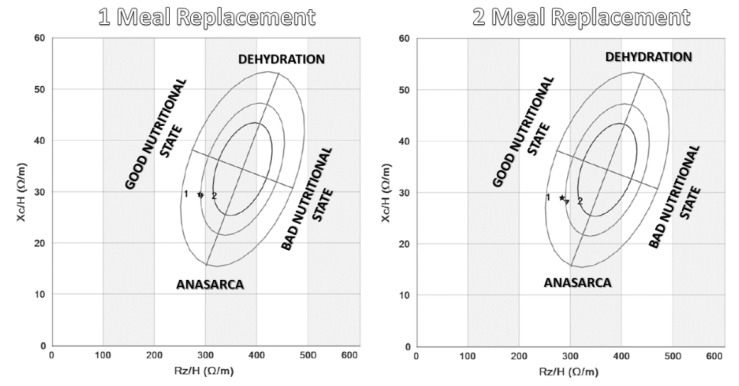
Changes in phase angle before (1) and three months after the intervention (2) in both randomized groups. The electrical parameters of resistance (Rz/H) and reactance (Xc/H) were directly assessed, and the alpha phase angle and impedance (Z) were estimated. Body composition was estimated, and vector impedance was assessed with Biva^®^ and Bodygram^®^ software (AKERN).

In the determination of the phase angle, a significant decrease was observed in the group of two meal replacements without significant differences between the groups (two meal replacements: 0.29 (1.08) vs. one meal replacement: 0.03 (1.01); *p* = 0.20) (Figure 3). This indicated the relative decrease of total mass and body water and a relative increase in fat-free mass. 

### 3.3. Cardiovascular Risk Factors:

**Figure 4:**
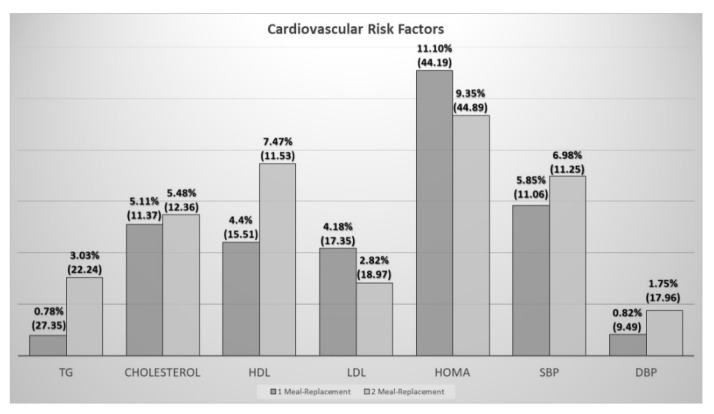
Comparison of changes over three months in cardiovascular risk factors (percentage of changes in the Y axis) and differences between the two groups of intervention. TG: triglycerides; HDL: high density lipoprotein; LDL: low density lipoprotein; SBP: systolic blood pressure; DBP: diastolic blood pressure.

When assessing blood pressure, no differences were found at the beginning between both groups, and the distribution of patients with arterial hypertension between groups was similar (Table 1). A decrease in systolic blood pressure was observed in both groups (Table 3); without significant differences between groups (one meal replacement: −8.65 (15.18) mmHg vs. two meal replacements: −10.47 (16.06) mmHg) (*p* > 0.05) (Figure 4). 

In the case of plasma lipids, a decrease in total cholesterol and HDL was observed in both groups. In the group of one meal replacement, it was also observed in LDL and in the group of two meal replacements in triglycerides (Table 4).

Finally, when we evaluated the parameters of glycemic control and insulin resistance, a significant decrease in glycemia and insulinemia was observed in the group of one meal replacement, while in the group of two, only a decrease in glycemia was observed. HOMA-IR was calculated for the assessment of insulin resistance, finding a significant decrease in both groups. No differences between groups were observed in relation to the alteration of the glycidic metabolism (Figure 4).

## 4. Discussion

In our study, the meal replacement diet strategy showed a decrease in weight, an improvement in the body composition profile with a relative decrease in the fat component, and an improvement in cardiovascular risk factors. These effects occurred independently of the group to which patients were randomized (one or two meal replacements).

The main characteristic of the patients studied was the diagnosis of osteoarthritis. In this type of patient, the body composition, especially in relation to the percentage of fat mass, was similar of our study. Thus, in a study conducted by Visser et al., a percentage of fat mass of 29% was observed in men and 43.3% in women [17]. Another study by Ertürk showed an increase in the amount of fat mass and a decrease in fat-free mass in patients with knee osteoarthritis compared to the healthy population [18].

The main objective variable in the study was the influence of the meal replacement diet on weight and BMI three months after the start of treatment. A significant decrease in weight was observed in both groups between 5 and 10% without differences between them. The effectiveness of meal replacement diets has been studied many times. The effect of these short-term diets (three months) is contrasted, and they handle similar weight losses. In a meta-analysis conducted in 2003, six controlled and randomized studies were evaluated in which it was observed that there was an average weight loss of between 6.19 and 6.50 kg (7% of total weight) compared to the control group where a loss of 3.23–3.99 kg was observed (4% of the initial weight) [7]. Another recent meta-analysis showed that the meal replacement diet had an adequate effect on weight loss both in the short and long term [19]. These results are in line with those obtained in our study.

When comparing with populations similar to ours, in a study carried out by De Luis in a population of patients with osteoarthritis, a greater weight loss was observed in the treatment group with a commercial diet versus the dietary advice [20] with a mean loss weight 7.7kg versus 3.92kg in the control group. 

The main difference with other studies of this type was the approach of randomly comparing a one meal replacement strategy versus a two meal replacement strategy. We saw a difference in all anthropometric and body composition parameters in both groups, but we have not seen differences between the two strategies. Regardless of the study group, having followed a diet strategy of meal replacement and the follow-up by a specialized dietary team probably influenced the observed effect on weight loss in both groups [21]. The importance of the lack of differences in these two approaches could help us to adapt the meal replacement strategy according to the probability of adherence in each patient. 

Waist and hip diameters decreased significantly after the intervention in the general sample. A decrease in the waist was observed in both groups without differences between them. When analyzing meal replacement diets versus hypocaloric diets, a greater decrease in waist diameter was observed in the replacement diet group [22,23]. On the other hand, in other studies, a similar effect was observed in the different therapeutic interventions [24,25]. In our group, when assessing based on having made one or two meal substitutions, a significant decrease in waistline was observed in both groups. Although a somewhat higher power was observed in the group that complied with the protocol, the difference in the variations of the two groups was not significant.

A significant decrease in the absolute values of all the variables was observed in the analysis of body composition. The general decrease in the different compartments was related to the effect of weight loss associated with diet. The predominance in the loss of fat mass was related to the mobilization of deposits associated with a hypocaloric diet. The decrease in the rest of the components was small in proportion to that of fat mass in both groups, a situation similar to that observed in other studies related to meal replacement diets [26]. On the other hand, the values relative to the total weight at the beginning and after three months of intervention were analyzed. In this case, a significant difference was also observed in all parameters, but the decrease in the percentage of fat mass was associated with a relative increase in fat-free mass. This situation indicated that, although there was a loss of total weight and of all the components of the body composition, the loss was of greater magnitude in the fat component while maintaining the percentage of fat-free mass. This data showed us that, despite the caloric restriction, the maintenance of an adequate protein intake could help prevent an excess of muscle loss, which could enhance the sarcopenia situation detected in most patients. The predominant decrease in fat mass with maintenance or the relative increase in the percentage of muscle mass and fat mass has been observed in different studies with hyperproteic diets replacing one or more meals [10,27] or in studies replacing a meal versus a usual diet [28].

Diet by meal replacement can influence body composition, at least similar to the normal diet, while maintaining similar percentages of macronutrients [29]. This is the objective in different studies that analyze the composition of the preparations with a greater protein intake associated with a greater loss of fat mass [10,25].

When we start low-calorie diets in the elderly population, one of the biggest concerns is the negative influence we can have on muscle mass [30]. In our population, this situation was enhanced by the decrease in mobility and, therefore, in the physical activity that these patients have. To assess the relative change in body composition in relation to the possible negative effects of weight loss in the elderly patient, the previously defined index with the muscle and fat component was analyzed. A significant change was observed with an increase in both groups. The improvement in this index and therefore in the diagnosis of sarcopenic obesity can be associated with weight loss along with the increase in protein intake, a profile that has been observed as the most appropriate in weight loss diets [31] and that is reflected in the ESPEN (European Society of Clinical Nutrition and Metabolism) recommendations on protein consumption and exercise in the elderly [32].

Regarding the parameters related to cardiovascular risk, there was an improvement in the variables related to glycidic metabolism (insulin, baseline glycemia, and HOMA-IR), probably related to weight loss. When assessing the use of meal replacement diets in diabetic patients, a decrease in resistance, glycated hemoglobin, and therefore, insulin needs has been observed [33]. No differences were observed between both groups (one or two substitutions) unlike the study by Leader et al., in which comparing the replacement of one versus two meals with commercial preparations in diabetic patients showed a better control in HbA1c in the group of two substitutions [34]; or the study by Kempf et al., in which the three meal substitution strategy was more effective in glycemic control [35].

A decrease in all lipid parameters was observed except for triglycerides in the one meal replacement group, probably related to the intake of more saturated fat in the food that they did not replace and in the LDL in the substitution group due to possible variations in the intermediate intake and the influence of hypolipidemic treatment itself. The use of meal replacement diets has shown in multiple studies an improvement in the lipid profile (decrease in total cholesterol, LDL, and triglycerides) with a decrease in HDL cholesterol similar to our study [23,36,37].

When comparing blood pressure according to the study group, a decrease in systolic blood pressure was observed in both groups without differences between them. This effect is probably related to weight loss, which is in line with the multiple guidelines for the treatment of hypertension and obesity, in which weight loss is highly recommended for control; as a 5% decrease in weight can achieve a 2–3 mmHg decrease in blood pressure [38].

The effect on diastolic tension was not significant in any of the cases. According to what we know in relation to the treatment of hypertension, the effect of diet compared to pharmacological treatment on this parameter is more discreet, and yet, it has great importance in the development of cardiovascular disease [39].

The main limitation of the study was its design for evaluating short-term changes. It would be interesting to evaluate the effect of these two diet methods in the long term. The study of cardiovascular risk factors was carried out using subrogated parameters. The most appropriate would have been the evaluation of the rate of vascular events, but this requires evaluation of studies of a very long duration and maintenance of these therapies for longer. The study of body composition by bioimpedanciometry in the patient with obesity may have some limitations. These limitations have been attempted to be controlled by evaluating the phase angle.

## 5. Conclusions

The main conclusions obtained from the present study were that in patients with obesity and osteoarthritis, the strategy of one meal replacement versus two meal replacements showed a significant decrease in weight and fat mass without differences between the two groups. The meal replacement strategy achieved a decrease in lipid parameters, glucose metabolism parameters, and systolic blood pressure regardless of the group to which they were assigned (one or two meal substitutions).

The use of the one meal replacement strategy appears to obtain similar results as that of two the meal replacement strategy and may achieve better adherence in these patients. 

## Figures and Tables

**Table 1:** Composition of the two randomized interventions: 1 meal replacement and 2 meal replacements.

	1 MEAL REPLACEMENT		2 MEAL REPLACEMENTS	
	MEN	WOMEN	MEN	WOMEN
Caloric Value (kcal)	1152.2	1021.4	1192.7	1035.9
Proteins (g (%TCV))	72 (25%)	64.2 (25%)	71.9 (24%)	64.4 (25%)
Lipids (g(%TCV))	31 (24%)	19.8 (17%)	19.9 (15%)	19.1 (17%)
*Cholesterol (mg)*	21.1	23.1	22.9	21
Carbohydrates (g(%TCV))	146.3 (51%)	146.6 (57%)	181.5 (61%)	151.6 (59%)
*Fiber (g)*	16.2	15.8	19.6	15.9

TCV: Total Caloric Value.

**Table 2:** Epidemiological variables, anthropometry, body composition, and cardiovascular risk factors before the intervention.

VARIABLES	TOTAL	1 MEAL REPLACEMENT *n* = 52	2 MEAL REPLACEMENTs *n* = 60	*p*-Value
Sex (% Women)	72.3	67.3%	76.7%	0.27
Age (years)	61.02 (11.03)	60.58 (11.21)	61.40 (10.95)	0.69
Osteoarthritis (%) (Knee/Hip/Spine)	79.5/6.3/14.3	78.8/7.7/13.5	80/5/15	0.83
Weight (kg)	101.4 (13.9)	100.9 (13.8)	101.8 (14.3)	0.75
BMI (kg/m^2^)	39.56 (5.22)	38.89 (5.11)	40.15 (5.27)	0.21
Waist (cm)	117.9 (13.6)	115.6 (17.2)	120.1 (9.0)	0.17
Hip (cm)	124.2 (15.1)	122.3 (19.1)	125.9 (10.4)	0.41
BIA Fat Mass (%)	45.59 (7.45)	44.79 (7.77)	46.28 (7.16)	0.29
BIA Fat-Free Mass (%)	54.29 (7.34)	55.10 (7.64)	53.58 (7.05)	0.27
BIA Phase Angle (^o^)	5.81 (1.15)	5.77 (1.13)	5.85 (1.17)	0.73
FMI (kg/m^2^)	18.38 (4.29)	17.69 (4.45)	18.97 (4.09)	0.12
FFMI (kg/m^2^)	21.37 (3.47)	21.43 (2.85)	21.33 (3.96)	0.88
Hypertension (%)	50%	52.9%	48.3%	0.63
SBP (mmHg)	133 (15)	132 (14)	134 (15)	0.51
DBP (mmHg)	81 (8)	80 (6)	82 (9)	0.12
Dyslipidemia (%)	8.9%	5.8%	11.7%	0.27
Triglycerides (mg/dL)	129 (50)	122 (45)	136 (53)	0.17
Total Cholesterol (mg/dL)	191 (34)	194 (33)	189 (35)	0.50
HDL (mg/dL)	53 (12)	52 (13)	54 (11)	0.55
LDL (mg/dL)	113 (29)	117 (27)	109 (31)	0.13
Diabetes Mellitus (%)	8%	3.8%	11.7%	0.13
Fasting Blood Glucose (mg/dL)	109 (26)	110 (31)	109 (22)	0.83
Insulin mU/mL	19.37 (15.11)	19.18 (12.28)	19.53 (17.33)	0.90
HOMA-IR	5.03 (3.52)	5.31 (4.01)	4.79 (3.04)	0.70

BMI: body mass index; BIA: bioimpedanciometry; FFMI: fat-free mass index; SBP: systolic blood pressure; DBP: diastolic blood pressure; HDL: high density lipoprotein; LDL: low density lipoprotein. *p*-value (differences between variables of two intervention arms). Values are represented as the mean (standard deviation) for continuous variables and the percentage of the reporting population for qualitative variables. HOMA-IR: Homeostatic Model Assessment – Insulin Resistance.

**Table 3:** Changes in anthropometric and body composition parameters before and after 3 months of the intervention in both groups (1 meal replacement vs. 2 meal replacements).

VARIABLES	1 MEAL REPLACEMENT *n* = 52				2 MEAL REPLACEMENTS *n* = 60			
	START	3 MONTHS	Change	*p*	START	3 MONTHS	Change	*p*
Weight (kg)	100.9 (13.8)	93.0 (15.1)	7.9 (5.7)	<0.01	102 (14.3)	93.1 (13.6)	8.7 (3.6)	<0.01
BMI (kg/m^2^)	38.72 (5.23)	36.18 (5.11)	2.54 (3.04)	<0.01	39.96 (5.13)	36.63 (4.79)	3.34 (5.95)	<0.01
Waist (cm)	115.5 (17.4)	110.7 (10.1)	4.9 (16.3)	0.04	120.2 (9.1)	113.7 (9.8)	6.5 (6.3)	<0.01
Hip (cm)	122.3 (19.1)	120.1 (16.7)	2.2 (19.8)	0.43	125.9 (10.5)	119.1 (10.3)	6.8 (5.1)	<0.01
Fat Mass (%)	44.79 (7.77)	42.71 (8.64)	2.07 (4.3)	<0.01	46.28 (7.16)	43.14 (8.5)	3.14 (5.60)	<0.01
Fat-Free Mass (%)	55.10 (7.64)	58.67 (8.50)	3.57 (4.61)	<0.01	53.58 (7.05)	55.72 (8.91)	2.14 (4.45)	<0.01
Phase Angle (^o^)	5.77 (1.13)	5.74 (0.98)	0.03 (1.01)	0.82	5.86 (1.18)	5.57 (0.85)	0.29 (1.08)	0.04
FMI (kg/m^2^)	17.69 (4.45)	15.54 (4.42)	2.15 (1.45)	<0.01	18.97 (4.09)	16.19 (4.16)	2.78 (2.55)	<0.01
FFMI (kg/m^2^)	21.43 (2.84)	20.91 (2.65)	0.52 (0.83)	<0.01	21.33 (3.96)	20.44 (3.71)	0.89 (0.98)	<0.01
FFM/FM	1.32 (0.53)	1.48 (0.59)	0.16 (0.16)	<0.01	1.22 (0.43)	1.40 (0.59)	0.17 (0.34)	<0.01

BMI: body mass index; FFMI: fat-free mass index; FMI: fat mass index; FFM/FM: fat-free mass/fat mass. *p*-value (differences between start and 3 months). Values are represented as the mean (standard deviation) for continuous variables and the percentage of the reporting population for qualitative variables.

**Table 4:** Changes in cardiovascular risk parameters before and 3 months after intervention (1 meal replacement vs. 2 meal replacements).

VARIABLES			1 MEAL REPLACEMENT			2 MEAL REPLACEMENTS		
	START	3 MONTHS		*p*	START	3 MONTHS		*p*
SBP (mmHg)	131.92 (14.48)	123.26 (12.52)	8.65 (15.18)	<0.01	133.77 (14.99)	123.30 (10.91)	10.47 (16.06)	<0.01
DBP (mmHg)	79.56 (6.33)	78.61 (6.86)	0.94 (7.5)	0.37	81.67 (8.91)	79.85 (14.03)	2.01 (14.94)	0.30
Triglycerides (mg/dL)	121.59 (44.73)	113.98 (39.03)	7.61 (36.33)	0.14	136.15 (53.53)	125.75 (40.11)	10.41 (32.75)	0.02
Total Cholesterol (mg/dL)	192.33 (31.98)	181.07 (29.59)	11.26 (21.45)	<0.01	189.17 (35.61)	177.44 (34.52)	11.73 (24.67)	<0.01
HDL (mg/dL)	51.43 (12.62)	48.61 (12.20)	2.82 (8.01)	0.01	53.05 (10.89)	48.30 (7.64)	4,75 (6.81)	<0.01
LDL (mg/dL)	116.63 (26.88)	109.75 (25.06)	6.88 (18.43)	0.01	108.86 (31.09)	103.98 (31.61)	4.87 (19.83)	0.06
Blood Plasma Glucose (mg/dL)	110.41 (30.85)	104.53 (20.72)	5.88 (15.05)	<0.01	109.15 (22.13)	99.84 (19.69)	9.31 (21.32)	<0.02
Insulin (U/mL)	19.19 (12.28)	15.16 (8.01)	4.02 (7.91)	<0.01	19.74 (17.77)	15.95 (10.30)	3.79 (17.21)	0.11
HOMA-IR	5.37 (4.02)	3.95 (2.27)	1.42 (2.81)	<0.01	4.81 (3.12)	3.91 (2.58)	0.89 (2.33)	<0.01

SBP: systolic blood pressure; DBP: diastolic blood pressure; HDL: high density lipoprotein; LDL: low density lipoprotein.
